# Tissue-engineered tubular substitutions for urinary diversion in a preclinical rabbit model

**DOI:** 10.3389/fmed.2025.1616977

**Published:** 2025-06-26

**Authors:** Qianliang Wang, Qingling Liu

**Affiliations:** ^1^Department of Urology, Affiliated Hospital of Guangdong Medical University, Zhanjiang, China; ^2^Department of Record Room, Affiliated Hospital of Guangdong Medical University, Zhanjiang, China

**Keywords:** tissue engineering, adipose-derived stem cells, smooth muscle cells, decellularized fish swim bladder, epithelium, urinary diversion

## Abstract

**Objective:**

To develop and evaluate tissue-engineered tubular constructs using homologous adipose-derived stem cells (ASCs), smooth muscle cells (SMCs), and decellularized fish swim bladder (DFSB) matrix for urinary diversion in a rabbit model.

**Methods:**

Rabbit ASCs and SMCs were isolated and expanded *in vitro*; cultured cells were seeded onto bilateral surfaces of DFSB scaffolds followed by 7-day incubation; cell-seeded matrices were shaped into tubular constructs; constructs underwent 2-week *in vivo* pre-vascularization within omental pouches. Experimental group rabbits (*n*=24) underwent complete bladder resection with replacement by pre-vascularized constructs, while control group (*n*=6) received identical implantation of acellular DFSB tubes. Histological evaluations were conducted at postoperative weeks 2, 4, 8, and 16; intravenous urography (IVU) was performed at 16-week endpoint.

**Results:**

All experimental animals survived until scheduled sacrifice with histological evidence of: (1) luminal multilayer urothelium, (2) organized smooth muscle tissue on abluminal surfaces, and (3) construct-wide neovascularization of varying diameters; IVU confirmed absence of urinary leakage, stricture, or obstruction. Conversely, all control animals died within 2 weeks post-operation; autopsy revealed urine leakage, extensive scar formation, and severe inflammation as mortality causes.

**Conclusion:**

Tissue-engineered tubular constructs fabricated from homologous ASCs, SMCs, and DFSB scaffold demonstrate feasibility as a viable urinary diversion alternative in rabbit models, showing functional tissue regeneration and superior outcomes versus acellular controls.

## Introduction

Congenital disorders or acquired pathologies can cause significant bladder tissue damage, often requiring complex surgical reconstruction. Currently, the ileal conduit remains the preferred urinary diversion method following cystectomy despite well-documented disadvantages (1). This approach carries substantial risks of complications (2, 3), including dual surgical trauma, chronic urinary tract infections, electrolyte imbalances, and problematic mucus secretion-all significantly compromising patients’ quality of life (4). Consequently, developing alternative methods for urinary diversion to mitigate these complications is critically important.

Tissue engineering, an advancing field, offers promising solutions to current therapeutic challenges in urinary diversion for patients undergoing radical cystectomy. This approach harnesses autologous cells and biocompatible materials to regenerate functional tissues and organs. Within the urinary system, the specialized urothelium forms an essential blood-urine barrier protecting underlying tissues (5). Successful bladder reconstruction in both animal models and humans fundamentally depends on establishing this epithelial layer (6). However, patients with malignant bladder disease often lack sufficient healthy urothelium for harvesting.

Adipose-derived stem cells (ADSCs) present a promising solution. Easily harvested from abundant adipose tissue with minimal risk, ADSCs possess self-renewal capacity and multilineage differentiation potential - key attributes for bladder regeneration. Notably, research demonstrates ADSCs’ ability to differentiate into epithelial lineages. When co-cultured with urothelial cells, they express urothelial-specific proteins uroplakin Ib and II (7, 8). Furthermore, ADSCs secrete bioactive factors that promote vascularization in ischemic tissues (9).

Decellularized fish swim bladder (DFSB) scaffolds provide an excellent regenerative platform. By removing cellular components while preserving the native extracellular matrix (ECM) - rich in collagen, elastin, and proteoglycans - DFSB retains critical bioactivity to support cell growth and differentiation (10).

Adequate vascularization remains a fundamental challenge in tissue transplantation, as insufficient blood supply can trigger inflammation, ischemia, and fibrosis. The greater omentum, renowned for its potent angiogenic properties, serves as an ideal *in vivo* bioreactor for prevascularizing engineered tissues (11).

In this study, we isolated and expanded rabbit ADSCs and smooth muscle cells (SMCs) *in vitro*. These cells were then seeded onto opposing surfaces of DFSB scaffolds to create tissue-engineered tubular substitutes (TETS). Following *in vitro* maturation, TETS were implanted within the greater omentum for 2 weeks to establish vascular networks. We subsequently evaluated the feasibility of using these prevascularized TETS as urinary diversion conduits in a rabbit cystectomy model.

## Materials and methods

### Animals

All animal experiments and handling procedures strictly adhered to the guidelines established by the Institute of Laboratory Animal Resources at the Affiliated Hospital of Guangdong Medical University. This research protocol underwent formal review and received full approval from the hospital’s Laboratory Animal Ethics Committee (Approval Number: GDY2302199). Male New Zealand White rabbits (body weight range: 2.0–2.5 kg) were procured from the Experimental Animal Center of Guangdong Medical University. Animals were housed under standardized, environmentally controlled conditions within the Medical Department’s Animal Care Facility at Guangdong Medical University. Throughout the study period, rabbits had ad libitum access to food and water. This study was conducted and reported in strict accordance with the ARRIVE (Animal Research: Reporting of *In Vivo* Experiments) guidelines.

### Isolation and culture of ADSCs and SMCs

Adipose-derived stem cells (ADSCs) were isolated from subcutaneous adipose tissue harvested from New Zealand White rabbits. Under aseptic conditions and general anesthesia, approximately 10 g of adipose tissue was surgically dissected from the inguinal region. Tissue specimens were immediately placed in phosphate-buffered saline (PBS) supplemented with antibiotics (penicillin/streptomycin) for transport and processing. ADSC extraction was performed using enzymatic digestion with 0.1% type I collagenase. The adipose tissue underwent sequential preparation: (1) Triple-washing in PBS to remove blood contaminants. (2) Mechanical mincing into <1 mm^3^ fragments. (3) Digestion in collagenase solution at 37°C for 60 min with periodic agitation. Digestion was terminated by adding Dulbecco’s Modified Eagle Medium (DMEM) containing 10% fetal bovine serum (FBS) and dual antibiotics (150 U/mL penicillin and 150 U/mL streptomycin). The cellular suspension was centrifuged at 805 g for 10 min. The resulting stromal vascular fraction (SVF) pellet was resuspended in complete growth medium (DMEM with 10% FBS and antibiotics) and plated in 10 cm culture dishes. The specific protocol for isolating adipose-derived stem cells (ADSCs) is described in the Methods section (12). After 24-h incubation at 37°C in 5% CO₂, the medium was replaced to eliminate non-adherent cells and erythrocyte debris. Cultures were maintained with medium changes every 72 h. ADSCs between passages 3–5 were utilized for subsequent experiments, ensuring consistent cell phenotype and functionality.

Bladder smooth muscle cells (SMCs) were isolated and expanded using an established tissue explant technique (13). Briefly, partial cystectomies were performed on ten New Zealand White rabbits, with full-thickness muscular biopsies harvested from the bladder wall. Primary cell cultures were initiated by explanting minced tissue fragments (<2 mm^3^) into 10-cm culture dishes containing Dulbecco’s Modified Eagle Medium (DMEM) supplemented with 10% fetal bovine serum (FBS). Cultures were maintained at 37°C in a humidified 5% CO₂ incubator. Following 7 days of primary outgrowth, confluent cells were dissociated using 0.25% trypsin–EDTA, washed twice in phosphate-buffered saline (PBS), and reseeded into new culture vessels at appropriate densities. SMCs between passages 3–5 ensuring phenotypic stability while avoiding senescence - were utilized for all downstream applications.

### Identification of ADSCs and SMCs

Flow cytometry was employed to characterize the immunophenotypic profile of adipose-derived stem cells (ADSCs) using established mesenchymal stem cell markers. Third-passage ADSCs (approximately 2 × 10^6^ cells) were enzymatically detached, washed twice with phosphate-buffered saline (PBS), and resuspended in 100 μL staining buffer. Cells were incubated with fluorescein isothiocyanate (FITC)-conjugated monoclonal mouse anti-rabbit primary antibodies targeting CD90, CD44, CD34, and CD45 (all purchased from Abcam, Cambridge, UK) at manufacturer-recommended concentrations for 45 min at 4°C protected from light. Following two additional PBS washes, cells were resuspended in 500 μL ice-cold PBS and analyzed immediately using a FACSCalibur flow cytometer (BD Biosciences, Franklin Lakes, NJ, USA). Isotype-matched antibodies served as negative controls. A minimum of 10,000 events were acquired per sample, with data analysis performed using CellQuest Pro software (BD Biosciences). Concurrently, smooth muscle cell (SMC) differentiation was confirmed through immunofluorescence staining for *α*-smooth muscle actin (α-SMA). Cultured SMCs were fixed in 4% paraformaldehyde, permeabilized with 0.1% Triton X-100, and incubated with primary anti-α-SMA antibody (1:200 dilution) followed by appropriate Alexa Fluor-conjugated secondary antibodies. Nuclei were counterstained with DAPI (4′,6-diamidino-2-phenylindole), with fluorescent images captured using a confocal microscope.

### Preparation of decellularized fish swim bladder (DFSB)

Decellularized scaffolds were prepared from swim bladders harvested from *Cyprinus carpio* (n = 50). Tissues underwent sequential processing as follows: (1) Initial stabilization: Specimens were rinsed in phosphate-buffered saline (PBS; pH 7.4) containing 0.1% w/v EDTA and 10 KIU/mL aprotinin (Trasylol®) to inhibit endogenous protease activity. (2) Osmotic treatment: Tissues were equilibrated in hypotonic Tris–HCl buffer (10 mM, pH 8.0) at 4°C for 24 h with gentle agitation. (3) Surfactant processing: Samples were transferred to 0.1% w/v sodium dodecyl sulfate (SDS) solution and incubated on an orbital shaker (50 rpm) at room temperature (22 ± 2°C) for 24 h. (4) Nuclease digestion: Following PBS rinses, tissues were treated with 10 mM Tris–HCl (pH 7.5) containing 2000 Kunitz units/mL deoxyribonuclease I (DNase I) at 37°C for 24 h with continuous agitation. (5) Lipid removal: Specimens were immersed in 4% w/v sodium deoxycholate with 0.1% w/v sodium azide and mixed at room temperature for 24 h. This cycle was repeated twice with fresh solution. After three final PBS washes under aseptic conditions, scaffolds were sterilized in 0.1% v/v peracetic acid for 2 h. The decellularized matrices were stored in antibiotic solution (100 U/mL penicillin, 100 μg/mL streptomycin, pH 7.2) at 4°C until characterization. Decellularization efficacy was validated through: (1) Histological analysis (H&E, Sirius Red, Masson’s trichrome staining). (2) Ultrastructural examination via scanning electron microscopy (SEM). (3) Quantitative assessment of residual nucleic acids (not specified in original). These complementary techniques confirmed cellular component removal while evaluating extracellular matrix preservation. Decellularized Fish Swim Bladder Fabrication Methodology followed established tissue-engineering standards (14, 15).

### Seeding cells onto the DFSB and producing TETSs

Adipose-derived stem cells (ADSCs) at passages 3–5 were enzymatically dissociated using trypsin to generate single-cell suspensions. These suspensions were subsequently seeded onto the luminal surface of the DFSB matrix at a density of 2 × 10^7^ cells/cm^2^ and cultured for 3 days. Concurrently, smooth muscle cells (SMCs) from passages 3–5 were similarly trypsinized and seeded onto the abluminal side of the same DFSB scaffold. Following cell seeding, the composite matrix was maintained in Dulbecco’s Modified Eagle Medium (DMEM) under standard culture conditions for 1 week, with the medium replaced daily to ensure nutrient availability and waste removal. Cellular proliferation and spatial distribution across the composite matrix were then assessed using hematoxylin and eosin (H&E) staining for histological evaluation and scanning electron microscopy (SEM) for ultrastructural analysis. After confirming ADSC confluency on the luminal surface, the cellularized DFSB was wrapped around an 10 French (Fr) guiding catheter to establish tubular geometry. The construct was then trimmedand sutured (5–0 chromic absorbable suture) to a standardized length of 6 cm, resulting in the generation of tissue-engineered tubular Substitutions (TETSs) for further application.

### Transferring TETSs into the omentum of rabbits

Under general anesthesia, 24 tissue-engineered tubular structures (TETSs) were surgically implanted into the greater omentum of rabbits for a 2-week *in vivo* maturation period prior to urinary diversion surgery. This omental wrapping technique was employed to exploit its rich vascularization and bioactive factors for enhancing graft neovascularization. Following retrieval, the TETSs were systematically evaluated through hematoxylin and eosin (H&E) staining for histological architecture assessment, alongside immunohistochemistry (IHC) targeting cytokeratin AE1/AE3 to detect urothelial cell regeneration and CD31 to quantify neovascularization density. Concurrently, as an additional control cohort, six tubular grafts fabricated from decellularized foreskin scaffolds (DFSB) without cell seeding underwent identical omental implantation procedures and 2-week incubation. This unseeded DFSB group served to distinguish scaffold-mediated tissue responses from active cellular contributions during remodeling.

### Implanting TETSs into the rabbits for urinary diversion

In the experimental group, rabbits were anesthetized via intravenous administration of 3% sodium pentobarbital (30 mg/kg). A 4-cm longitudinal midline incision was created superior to the pubic symphysis to access the abdominal cavity. Following exposure of the bladder and identification of bilateral ureters, complete cystectomy was performed. The urethral stump was ligated with 4–0 silk sutures, while the distal ureters were spatulated and anastomosed to the proximal ends of the tissue-engineered tubular structures (TETSs) using 8–0 polypropylene sutures in a simple continuous pattern. The TETSs were then tunneled through a peritoneal window and secured to the parietal peritoneum with interrupted 6–0 sutures, terminating at a pre-marked stoma site on the abdominal wall. To establish urinary drainage, a silicone catheter (10Fr) was fixed at the distal anastomosis site for 7 days, facilitating continuous urine diversion. In the control cohort (*n* = 6), identical surgical procedures were performed, substituting TETSs with unseeded DFSB matrix tubular grafts of equivalent dimensions. Maintain 28–30°C for 24 h post-op to prevent hypothermia suppressing metabolism. Keep humidity at 40–60% to avoid respiratory irritation. Isolate individually for 48 h to reduce aggression or wound licking. Use sterile soft bedding (e.g., paper-based), not wood shavings, to prevent wound adhesion. Record daily parameters and intervene immediately for abnormalities.

### Histologic analysis and intravenous urography assessment

Six rabbits per experimental group were humanely euthanized at predetermined endpoints of 2, 4, 8, and 16 weeks post-transplantation (*n* = 6/timepoint). The implanted tissue-engineered tubular structures (TETSs) were explanted for histomorphometric evaluation. An additional control cohort (*n* = 6 rabbits) implanted with unseeded grafts underwent identical analysis protocols. Following harvest, specimens were immersion-fixed in 10% neutral-buffered formalin for 48 h, then dehydrated through graded ethanol series and embedded in paraffin. Serial sections (5 μm thickness) were prepared for: Histopathological assessment: Hematoxylin and eosin (H&E) staining; Immunohistochemical (IHC) profiling: Urothelial differentiation: Anti-cytokeratin AE1/AE3 (broad-spectrum epithelial marker), anti-ZO-1 (tight junction protein), and anti-uroplakin IIIa (umbrella cell-specific glycoprotein); Muscular/angiogenic remodeling: Anti-*α*-smooth muscle actin (α-SMA; smooth muscle identification) and anti-CD31 (endothelial cell marker for neovascularization quantification). Functional integration validation was performed at the 16-week terminal endpoint via intravenous urography (IVU) using iopromide contrast agent (370 mgI/mL). Dynamic radiographs captured at 1, 5, and 15-min intervals assessed: Renal excretion kinetics, Ureteral patency, TETSs conduit architecture and Absence of contrast leakage/obstruction.

## Results

### Morphological characterization and identification of cells

**Figure 1:**
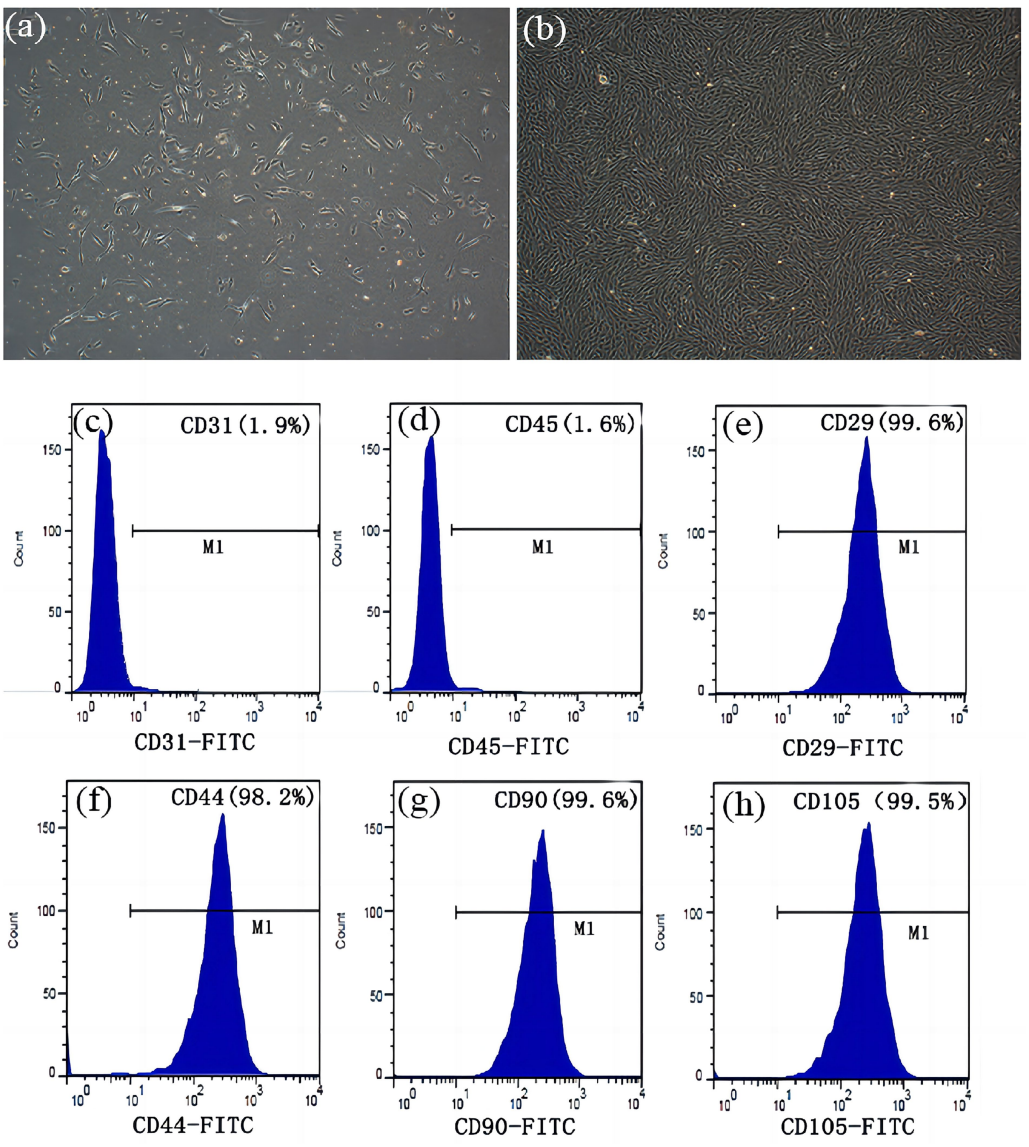
Morphological characterization and identification of ADSCs. **(a)** Primary culture after 3 days (x 100). **(b)** After incubation for 10 days, the cells proliferated rapidly and displayed a spindle fibroblastic appearance (x 100). There was no significant expression of **(c)** CD31 (1.90%), and **(d)** CD45 (1.60%). Flow cytometry analysis demonstrated expression of **(e)** CD29 (99.60%), **(f)** CD44 (98.20%), **(g)** CD90 (99.60%) and **(h)** CD105 (99.50%).

ADSCs were successfully isolated from rabbit adipose tissue via enzymatic digestion, and the primary cells exhibited typical spindle fibroblastic morphology (Figures 1a,b). To characterize the ADSC population, we performed flow cytometry to examine the phenotypic characteristics of mesenchymal stem cells. Passage 3–5 cells demonstrated high-level expression of CD29 (99.6%), CD44 (98.2%), CD90 (99.6%), and CD105 (99.5%), whereas negligible expression of CD31 (1.90%) and CD45 (1.60%) was observed. This result is consistent with established criteria for ADSC phenotypic markers (16) (Figures 1c–h).

**Figure 2:**
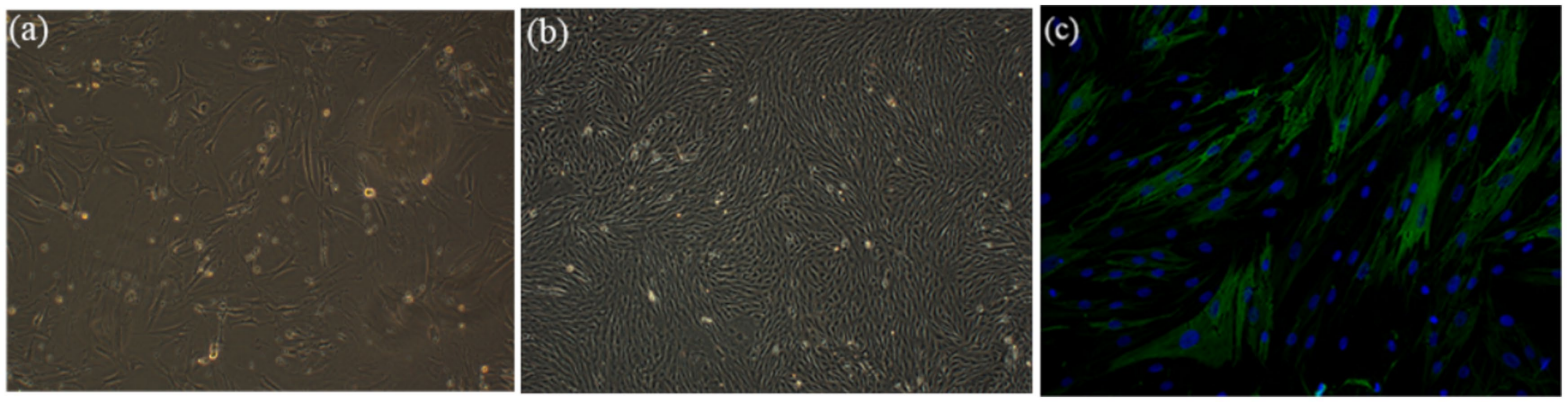
Morphological characterization and immunofluorescence staining of SMCs. **(a)** After 3 days of primary culture, the cells proliferated on the surfaces of Petri dish and reached about 30% confluence (x100). **(b)** After 7 days of culture, the cells reached 80–90% confluence and displayed a classic spindle-shaped morphology (x100). **(c)** a-SMA immunofluorescence staining (in green) of SMCs in combination with DAPI cell nuclei stain (in blue) (x200).

After 3 days of primary culture, smooth muscle cells (SMCs) demonstrated adherent proliferation on the Petri dish surfaces, reaching approximately 30% confluency (Figure 2a). By day 7, the cells achieved 80–90% confluency and exhibited characteristic spindle-shaped morphology as observed by phase-contrast microscopy (Figure 2b). Furthermore, immunofluorescence staining confirmed strong positive expression of *α*-smooth muscle actin (α-SMA) localized predominantly in the cytoplasm, indicating SMC contractile phenotype (Figure 2c).

### Characteristics of DFSB and matrix with seeded cells

**Figure 3:**
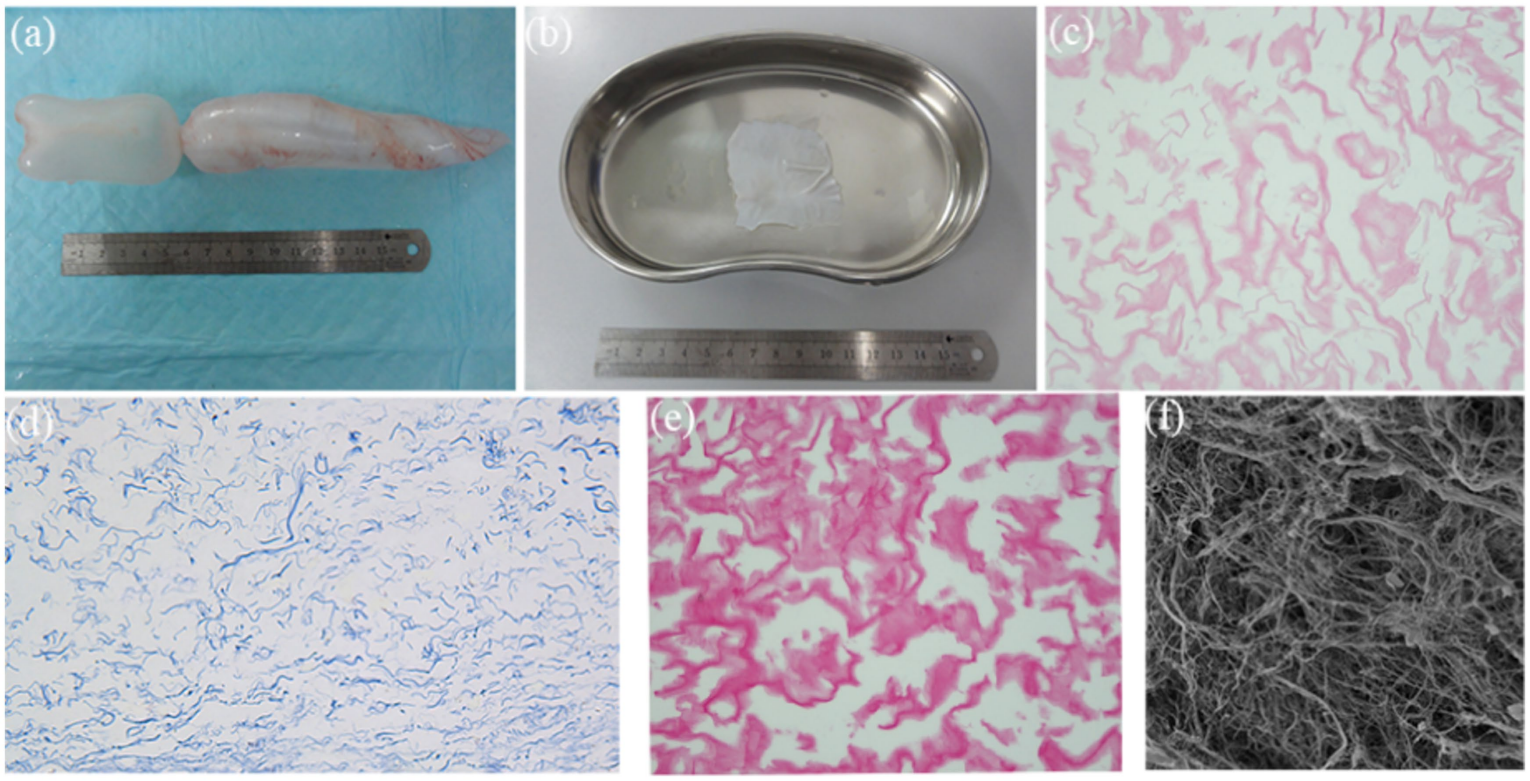
Characteristics of DFSB. **(a)** Fish swim bladder (gross appearance). **(b)** After decellularization procedure, the DFSB appeared as a translucent film (gross appearance). **(c)** H&E staining of DFSB (x400). **(d)** Masson’s trichrome staining of DFSB (x 400). **(e)** Sirius Red staining of DFSB (x400) **(f)** Scanning electron microscope of DFSB (x1000).

Fresh fish swim bladders (*Cyprinus carpio*) were procured from local seafood markets (Figure 3a). Following complete decellularization, the resulting decellularized fish swim bladder (DFSB) matrices exhibited translucent, sheet-like architecture (Figure 3b). Comparative histological analyses—including H&E staining, Masson’s trichrome staining, Sirius red staining, and scanning electron microscopy (SEM)—confirmed effective removal of cellular components in DFSB versus native tissue (Figures 3c–f).

**Figure 4:**
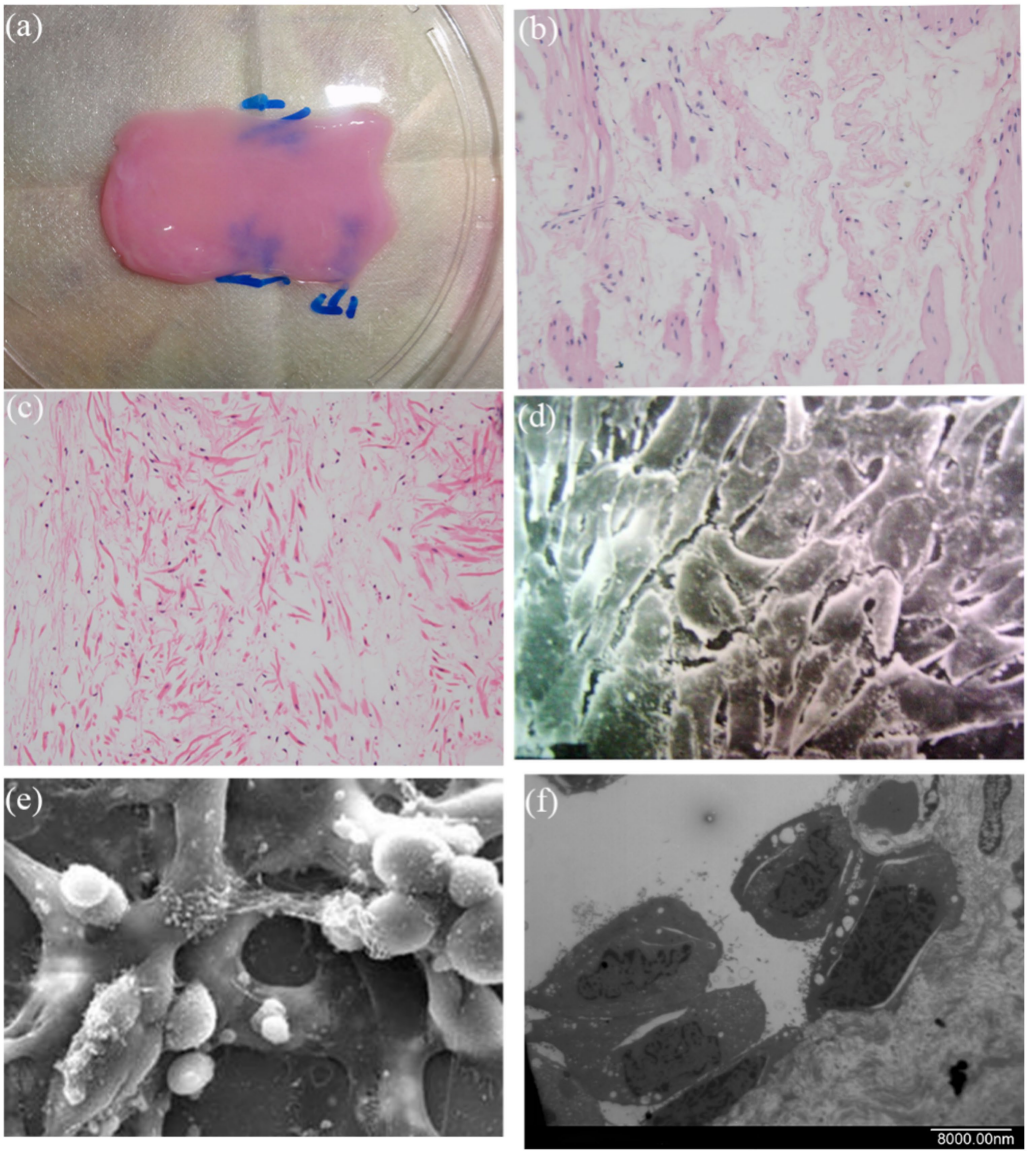
The feature of DFSB seeding. **(a)** DFSB with seeded cells. After seeding cells onto the DFSB, the cells fused well on the three-dimensional DFSB scaffold. **(b)** H&E staining of luminal side (x400). **(c)** H&E staining of outside side (x400). **(d)** Scanning electron microscope of luminal surface (x 2000). **(e)** Scanning electron microscope of outside surface (x2000). **(f)** Scanning electron microscope of luminal surface (x 2,500, longitudinal section).

After 7 days of ADSC and SMC seeding and incubation on decellularized fish swim bladder (DFSB) in DMEM, hematoxylin and eosin (H&E) staining and scanning electron microscopy (SEM) analysis of the composites confirmed effective cellular integration on bilateral DFSB surfaces (Figures 4a–f).

### Regeneration of epithelium and neovascularization after omental incubation

**Figure 5:**
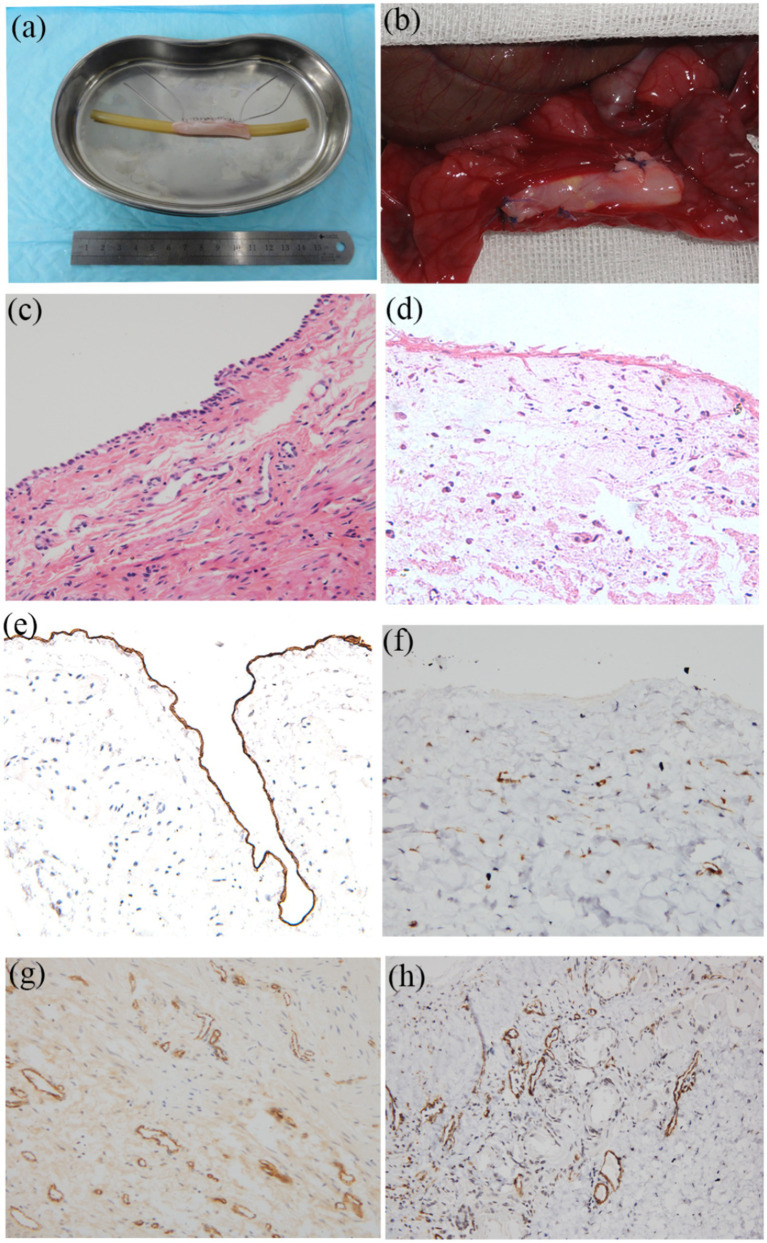
Regeneration of epithelium and neovascularization after omental incubation. **(a)** TETEs. **(b)** TETSs were wrapped in the omentum **(c)** H&E staining of TETSs (x 400). **(d)** H&E staining of the unseeded DFSB (x 400). **(e)** Anti-AE1/AE3 immunohistochemistry staining displayed a one-layer epithelium structure (x200). **(f)** Anti-AE1/AE3 immunohistochemistry staining showed no obvious epithelium in the unseeded DFSB (x 200). **(g)** Anti-CD31 immunohistochemistry staining revealed neovascularization of TETSs (x 200). **(h)** Anti-a-SMA immunohistochemistry staining revealed vascular walls in TETSs (x 200).

Following 7-day co-culture of cells with scaffolds, the cellularized matrix was circumferentially wrapped around a 10 Fr guiding catheter and trimmed to 6 cm in length to fabricate tissue-engineered tubular Substitutions (TETSs) (Figure 5a). After two-week omental wrapping (Figure 5b), H&E staining and immunohistochemistry (IHC) confirmed the presence of a confluent epithelial layer (anti-cytokeratin AE1/AE3) and robust neovascularization (anti-CD31/anti-*α*-SMA) within TETSs (Figures 5c,e,g,h). In contrast, no discernible epithelial structures were observed in unseeded DFSB controls (Figures 5d,f).

### Histological and intravenous urography evaluation

**Figure 6:**
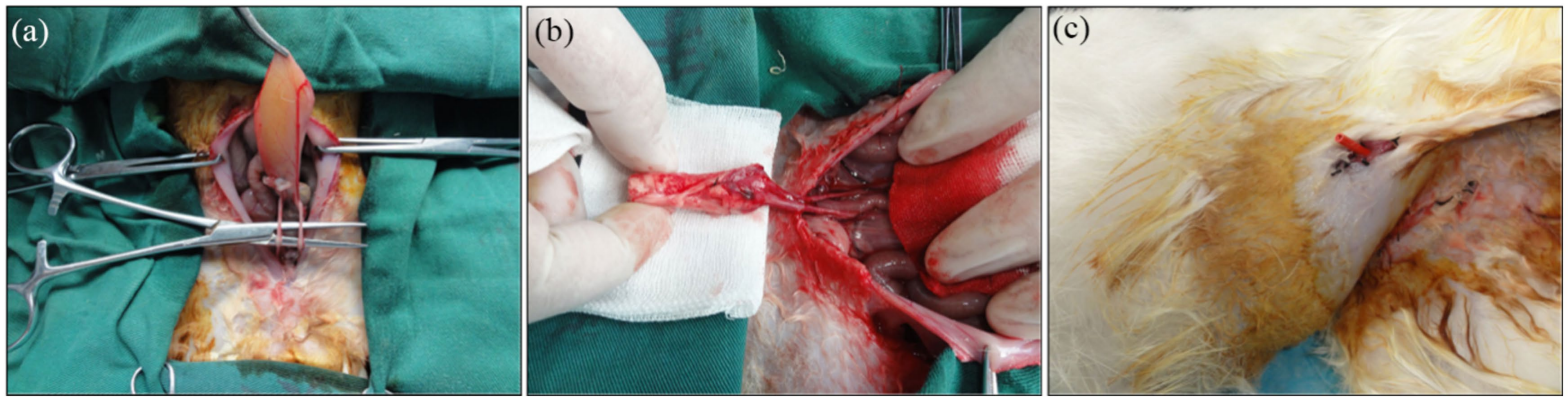
Urinary diversion in rabbits. **(a)** The bilateral ureters were dissociated. **(b)** Bilateral ureters were anastomosed to the TETSs. **(c)** Gross observation after Urinary diversion in rabbits.

**Figure 7:**
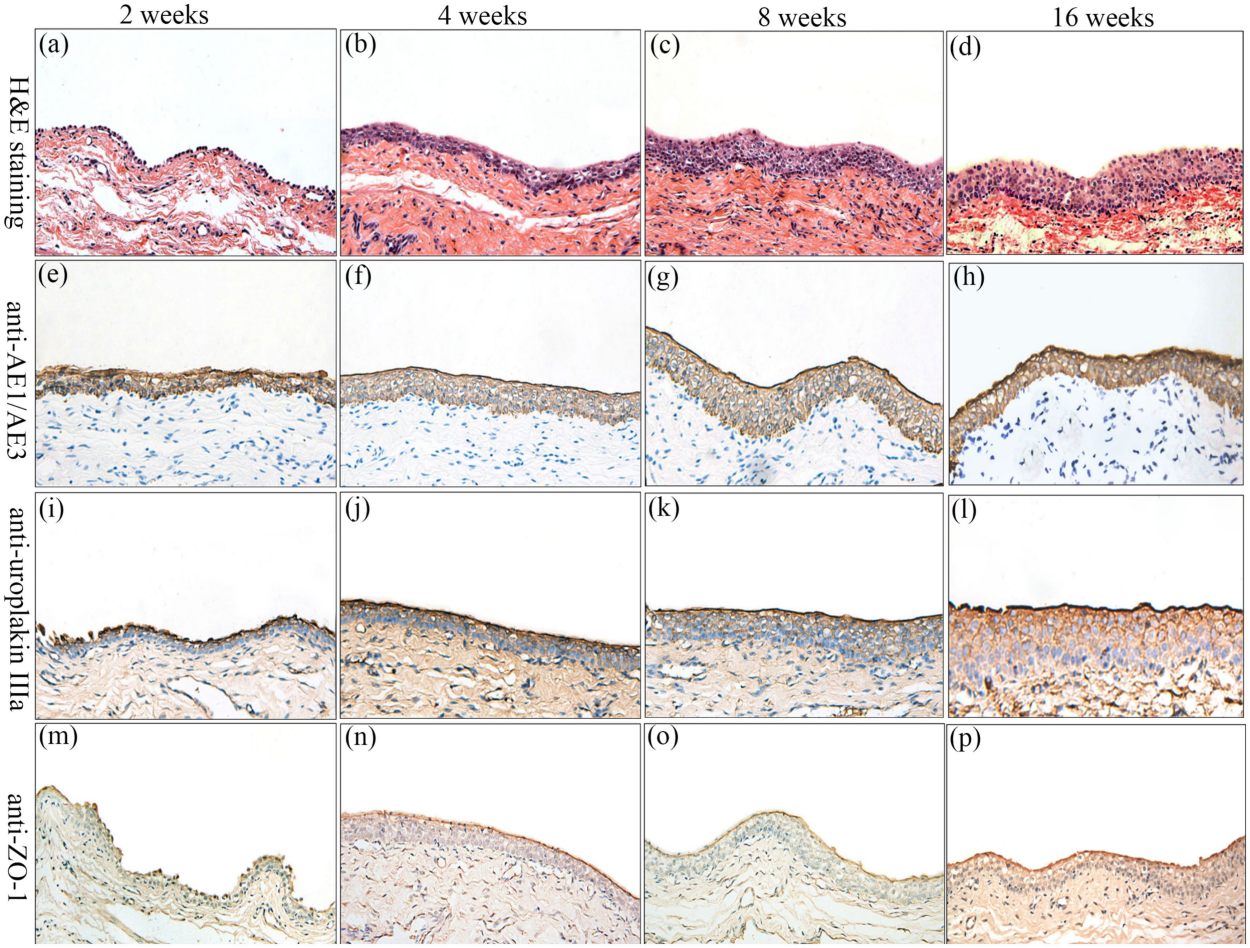
Histologic characteristics of TETSs in the experimental group after urinary diversion at 2, 4, 8, and 16 weeks. H&E staining **(a–d)** displayed the regeneration of epithelium layers of TETSs (x400). **(e–p)** Immunohistochemical staining of AE1/AE3, uroplakin IIIa, and ZO-1 revealed the regeneration of epithelium of TETSs, respectively (x 400).

**Figure 8:**
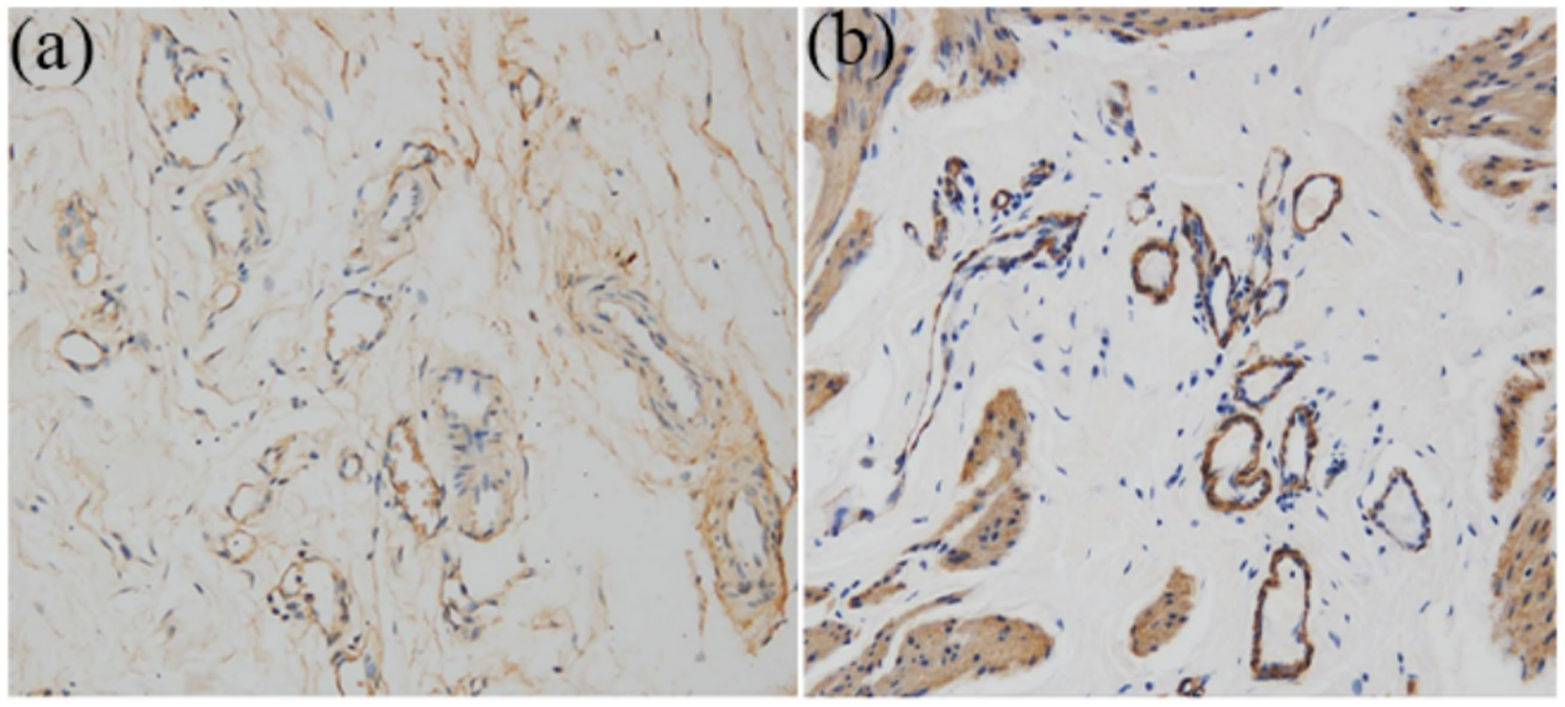
Immunohistochemistry analysis for regeneration of smooth muscle and neovascularization at 16 weeks (x 200). **(a)** Anti-CD31 antibody positive showed well-developed angiogenesis. **(b)** Anti-a-SMA antibody positive revealed vessel wall.

**Figure 9:**
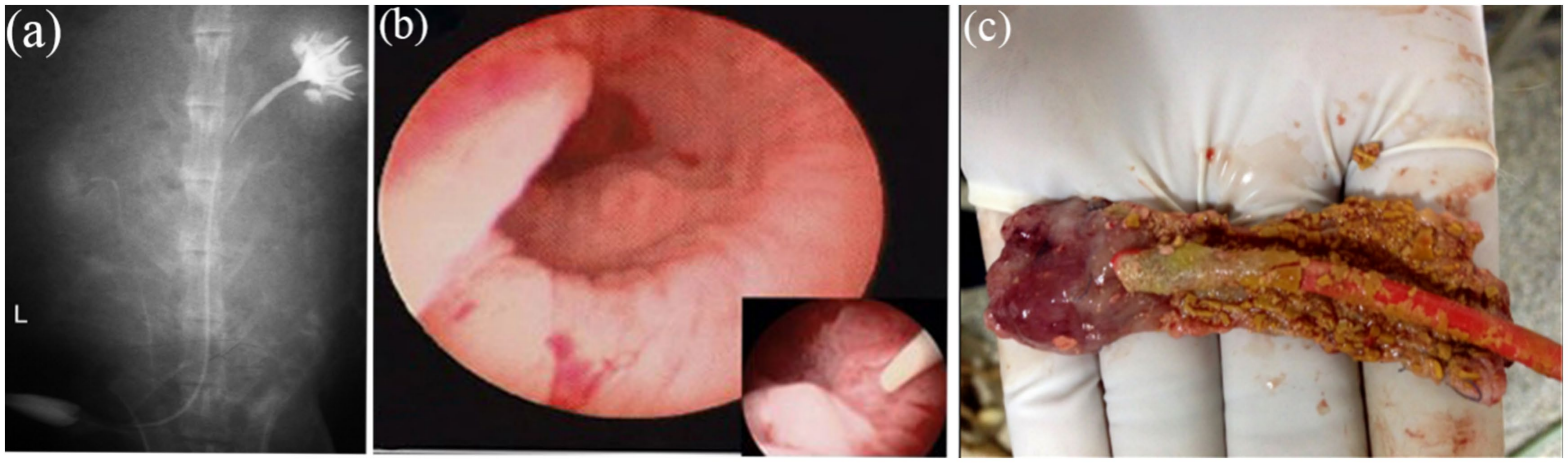
Intravenous urography observation. **(a)** Intravenous urography at16 weeks postoperatively showed urinary tract was unblocked and that no leakage, stricture, or obstruction occurred in the TETSs and bilateral ureters. **(b)** Cystoscopy showed that the surface of the bladder cavity were smooth. **(c)** In the control group, stone formation after urinary diversion at 2 weeks.

Surgical implantation of tissue-engineered tubular structures (TETSs) was performed in rabbits to establish urinary diversion (Figures 6a–c). In the experimental group, all animals survived until the predetermined endpoints. Histological evaluation of explanted composites demonstrated time-dependent epithelial maturation: At 2 weeks post-implantation, H&E staining revealed a thin, polarized epithelium covering the luminal surface (Figure 7a). By 4 weeks, progressive thickening of the transitional epithelial layer was observed (Figure 7b). From 8 to 16 weeks, both experimental groups exhibited continuous increase in epithelial stratification, with multilayered uroepithelium-like lining fully covering the graft lumen (Figures 7c,d). Immunohistochemical studies confirmed that the urothelial cells of the construct luminal sides were positively obtained with anti-AE1/AE3 (Figures 7e–h), anti-uroplakin IIIa (Figures 7i–l), and anti-ZO-1 antibodies (Figures 7m–p). At 16 weeks, all the TETSs were stained positively with anti-CD31 and anti-a-SMA antibodies (Figures 8a,b). In addition, intravenous urography revealed an unobstructed urinary tract with no evidence of leakage, stricture, or obstruction in the TETSs or ureter (Figure 9a). Cystoscopy demonstrated a smooth bladder cavity surface (Figure 9b). In stark contrast, all six animals receiving unseeded DFSB substitutes in the control group died within 2 weeks post-urinary diversion. Autopsy of these rabbits revealed urine leakage from tubular transplants, accompanied by marked inflammatory reactions, macroscopic tissue damage, and stone formation (Figure 9c).

## Discussion

Congenital and acquired bladder pathologies—including bladder exstrophy, neurogenic bladder, and muscle-invasive bladder cancer—often necessitate cystoplasty. Current surgical diversion options primarily comprise the ileal conduit, cutaneous ureterostomy, and orthotopic neobladder reconstruction. The current gold standard utilizes autologous intestinal segments for bladder augmentation or substitution. However, this approach carries significant morbidity, encompassing both immediate postoperative risks and long-term sequelae such as anastomotic complications, urinary calculi, excessive mucus secretion, bowel obstruction, metabolic derangements, and secondary malignancies (17, 18). Tissue-engineered urinary diversion represents a promising strategy to circumvent these intestinal complications and associated morbidities.

Two principal tissue engineering strategies offer viable approaches for urinary tract reconstruction: the use of acellular matrices alone, and acellular matrices seeded with cells. Both techniques represent potential alternatives for generating tissue-engineered constructs in diverse animal models. However, the unseeded acellular matrix approach faces significant limitations, notably inflammation and implant failure precipitated by direct exposure to the cytotoxic urinary environment following transplantation. In contrast, cell-seeded matrices circumvent these drawbacks and demonstrate superior regenerative potential in urological applications. Supporting this advantage, Yuan et al. (16) seeded human umbilical mesenchymal stem cells (HUMSCs) onto bladder acellular matrix (BAM) for canine bladder reconstruction. Comparative immunohistochemical analysis at 12 weeks post-implantation revealed that HUMSC-seeded BAM constructs (experimental group) significantly enhanced the development of a well-stratified urothelium and organized smooth muscle layers compared to unseeded BAM controls. Similarly, Orabi et al. (19) utilized autologous bladder epithelial and smooth muscle cells (SMCs) seeded onto preconfigured tubular acellular matrices to repair extensive urethral defects in canines, employing unseeded tubularized matrices as controls. Post-operative assessment via CT urethrography and histology demonstrated that cell-seeded matrices consistently facilitated the successful restoration of long urethral segments. In stark contrast, acellular collagen matrices alone resulted in inadequate tissue regeneration and frequent stricture formation.

Within the urinary system, the urothelium fulfills critical barrier and protective functions. It shields underlying acellular matrices and nascent tissues from the cytotoxic effects of urine while preventing urinary tract infections (20). These specialized functions rely on unique structural features of urothelial cells, particularly their apical plasma membrane and tight junction complexes (21). The integrity of the urothelial barrier fundamentally depends on the precise assembly of the apical plasma membrane. This membrane contains a distinctive lipid composition and epithelium-specific glycoproteins known as uroplakins (UPs). Mammalian urothelium expresses four major uroplakin subunits (UPIa, UPIb, UPII, and UPIIIa) that assemble into hexameric complexes, forming crystalline 16-nm particles that organize into impermeable two-dimensional plaques (22). As integral membrane proteins, these uroplakin plaques constitute a specialized permeability barrier against urinary solutes and pathogens. Crucially, uroplakins are synthesized abundantly only by terminally differentiated urothelial cells, establishing them as definitive differentiation markers (23). Complementing this barrier, tight junction proteins—particularly the multidomain scaffolding protein ZO-1—are essential for maintaining the high-resistance urothelial barrier. ZO-1 orchestrates tight junction assembly, forming membrane-associated complexes that critically regulate paracellular transport of ions, macromolecules, and immune cells (24).

For patients with urological malignancies or extensive tissue damage, organ biopsies often yield inadequate quantities of safe, expandable cells for transplantation. This critical limitation necessitates the exploration of alternative cell sources for urinary tissue engineering. Adipose-derived stem cells (ADSCs) have emerged as a highly promising candidate (25). Their advantages include relative abundance through minimally invasive harvest (liposuction), minimal donor-site morbidity, and inherent proliferative capacity. Critically, ADSCs possess demonstrable potential for epithelial differentiation under appropriate inductive conditions (26). *In vitro* studies using 3D culture systems have successfully induced rabbit ADSCs toward an epithelial lineage, resulting in cells exhibiting stratified epithelial-like morphology and expressing lineage-specific proteins (27). Subsequent validation confirmed the potential of these epithelial-differentiated rabbit ADSCs as substitutes for urothelial cells in urethral reconstruction (28). Further supporting this potential, Zhang et al. (29). demonstrated that human ADSCs, when exposed to a urothelial cell-conditioned medium or co-cultured with immortalized urothelial cells in a Transwell system, acquire a urothelium-like phenotype.

Optimal scaffolds for urinary diversion must exhibit biocompatibility to facilitate essential cellular processes—including adhesion, proliferation, and differentiation—while providing sufficient mechanical integrity to maintain graft structure. Critically, these scaffolds should not provoke adverse host responses, such as toxicity or chronic inflammation, post-implantation. Both naturally derived biomaterials and synthetic polymers are under investigation as potential urinary graft substitutes. Synthetic materials offer exceptional mechanical strength and can be precisely engineered to achieve tailored properties, such as elasticity, porosity, and degradation kinetics. Currently, extensively investigated synthetic polymers comprise poly(lactic-co-glycolic acid) (PLGA), poly(glycolic acid) (PGA), poly(*ε*-caprolactone) (PCL), and poly(lactic acid) (PLA). Among these, PGA has been widely studied as a representative synthetic polymer. Engelhard et al. (30) established that PGA scaffolds function as biodegradable matrices capable of promoting smooth muscle cell (SMC) regeneration. Complementing this, Kajbafzadeh et al. (31) engineered composite scaffolds through PGA integration with tissue-engineered pericardium, subsequently seeding them with urothelial cells and SMCs. Their findings demonstrated enhanced cellular adhesion and proliferation on these constructs, with *in vivo* studies confirming efficacy in facilitating bladder wall reconstruction. Despite advantages including scalable manufacturability and tunable physicochemical properties, synthetic polymers lack inherent bioinstructive cues to direct cellular growth and differentiation. Consequently, developing methodologies for effective incorporation of diverse bioactive factors constitutes a pivotal research focus for synthetic polymer-based systems (32). In contrast, Natural biomaterials are derived from native biological tissues and processed through physical or chemical methods. Currently, extensively studied natural biomaterials include bladder acellular matrix (BAM), small intestinal submucosa (SIS), amniotic membrane (AM), and acellular dermal matrix. SIS, a naturally derived extracellular matrix primarily composed of collagen, contains vascular endothelial growth factor within its structure as reported by Hodde et al. (33). Hurst et al. (34) detected multiple growth factors—including heparan sulfate proteoglycans, fibroblast growth factor, and vascular endothelial growth factor—in SIS scaffolds via immunohistochemical analysis, though transforming growth factor was not identified. SIS ranks among the most thoroughly investigated scaffold materials in both experimental and clinical models. Numerous studies demonstrate SIS’s capacity to support cellular proliferation, migration, and differentiation. Shukla et al. (35) isolated and cultured porcine bone marrow mesenchymal stem cells (BMSCs), inducing their differentiation toward urothelial smooth muscle cells (SMCs). These differentiated cells alongside undifferentiated BMSCs were subsequently seeded onto SIS for autologous bladder augmentation. Results confirmed SIS effectively supported cellular growth and differentiation, facilitating bladder tissue regeneration. Campodonico et al. (36) established that SIS maintains viability post-implantation, continuously supporting homologous urothelial cell growth and proliferation *in vitro* and in allogeneic models until scaffold degradation. Using nude mouse models, Zhang et al. (37) similarly validated SIS’s ability to promote cellular growth, differentiation, and tissue regeneration when seeded with SMCs and urothelial cells, providing experimental foundation for SIS-based seeded grafts in bladder reconstruction. However, conflicting findings have emerged. Feil et al. (38) reported insufficient urothelial cell viability on SIS, attributing observed cytotoxic effects in SIS-conditioned media to residual xenogeneic DNA. They concluded SIS is unsuitable as a substrate for urothelial cell growth. Ashley et al. (39) associated SIS with localized inflammatory responses, while Kropp et al. (40) observed host tissue contracture—factors that may limit SIS’s applicability in bladder tissue engineering. Decellularized fish swim bladder (DFSB) matrix naturally retains at least ten pivotal growth factors—such as VEGF, BMP4, PDGF-BB, and TGFβ1—which collectively establish a conducive microenvironment for cellular migration, proliferation, and differentiation (41). Concurrently, DFSB’s inherent porous 3D architecture maximizes its regenerative potential by promoting nutrient diffusion and cellular integration (42). In our study, DFSB served as a scaffold to construct TETSs. Expectedly, DFSB displayed excellent cell biocompatibility. Bhanu et al. Used both decellularized and fresh swim bladder for the repair of rabbit abdominal wall. The use of decellularized swim bladder resulted in less biochemical changes in the blood, less tissue reaction, lower antigenicity, and better tissue repair than when fresh swim bladder was used (43, 44). Baldursson et al. (45) further confirmed that the effect of swim bladder matrix on the wound healing speed may be better than that of decellularized porcine skin and cow leather products. Based on these studies, Jalali et al. first fabricated an acellular fish swim bladder (AFSBM), loaded it with exogenous HA as a carrier, and tested its wound repair ability using the rat back trauma model (46). Macroscopic and histological assessments of wound healing revealed that, compared with that of the other groups, the wound area of the AFSBM-HA group decreased rapidly, indicating faster wound healing. These findings all demonstrate that DFSB has high regenerative potential for cells and tissues. Moreover, the findings suggest that DFSB can be applied as a type of “off the shelf” scaffold material to load seeding cells for graft production, eventually hoping to promote host tissue regeneration.

Fibrotic encapsulation remains a major barrier to the advancement of tissue engineering, primarily attributed to microvascular ischemia-induced graft failure. Consequently, developing strategies to achieve rapid revascularization of tissue-engineered tracheal substitutes (TETSs) is imperative. Previous studies have established omentum wrapping as an effective method to enhance vascularization in tissue-engineered grafts (47, 48)—an approach further validated in our current study. Here, we employed omentum pre-wrapping to create an *in vivo* bioreactor environment that augments neovascularization within TETSs. After 2 weeks, the experimental group demonstrated significantly elevated epithelial structure formation and neovascularization density compared to controls.

Furthermore, intravenous urography (IVU) at the 16-week endpoint confirmed the absence of hydroureteronephrosis, urinary leakage, anastomotic stricture, or urinary tract obstruction. These findings demonstrate that TETSs effectively maintain functional urine drainage and preserve structural integrity in urinary diversion applications.

In summary, this study establishes the feasibility of constructing Tissue-Engineered Tubular Scaffolds (TETSs) using adipose-derived stem cells (ADSCs), smooth muscle cells (SMCs), and DFSB for urinary diversion in a rabbit model. Histological evaluations confirmed the development of a confluent, multilayered urothelial cell lining along the luminal surface of the grafts. This organized urothelium is critical for reestablishing the blood-urine barrier, thereby creating an impermeable microenvironment essential for subsequent tissue regeneration. Externally, SMCs demonstrated robust proliferation and differentiation, forming a structured muscular layer mirroring native anatomy. Furthermore, the omentum proved to be an effective in vivo bioreactor, significantly enhancing neovascularization and supporting graft regeneration. Collectively, these findings position TETSs as a promising emerging technology for clinical urinary diversion applications.

## Data Availability

The datasets presented in this study can be found in online repositories. The names of the repository/repositories and accession number(s) can be found in the article/supplementary material.
